# Address—Introductory to the Fourth Course of Lectures in the Atlanta Medical College

**Published:** 1858-06

**Authors:** W. F. Westmoreland

**Affiliations:** Professor of the Principles and Practice of Surgery


ATLANTA

Medical and Surgical Journal.

Vol. III.] JUNE, 1858. [No. X.

### ORIGINAL COMMUNICATIONS.

### ARTICLE 1.

*Address—Introductory to the Fourth Course of Lectures in the Atlanta Medical College.* By W. F. Westmoreland, M. D., Professor of the Principles and Practice of Surgery.

Gentlemen of the Medical Class:

I have been selected by my colleagues to welcome you to our halls; and while I deeply feel the compliment thus conferred, I no less feel my inability to give such a greeting as I would have you receive—such as would have been extended, had some other member of the Faculty been selected. Should I fall short of your expectations, and fail to give you that warm and cordial greeting which you, of right, should expect, do not attribute it to an absence of a proper feeling upon my part, for my heart is this hour full to overflowing with that unspeakable welcome, which I know swells the breast of every member of the body I represent.

Although we meet many of you to-day for the first time, yet we do not meet you as strangers, but as brothers—co-laborers in the same great work, with one mind, one hope, one aspiration; and as such, welcome you to our halls, our hearts and our homes. But a few days ago the most of you were surrounded by kindred and friends, in the full enjoyment of the precious privileges of the family circle. With a mother's prayer and a father's blessing, you left the paternal roof, and are here to-day to link your destinies with ours. Think not then, gentlemen, that you are as strangers in a strange land, but be assured that you are among those who heartily greet your coming, and welcome your arrival with the best feelings of their hearts. Yes, you are in the midst of those who will leave nothing undone during your sojourn in our city, that will contribute to your comfort or happiness; and who, we hesitate not to say, will be to you as father and friends, rejoicing with you in your success, and sympathizing with you in your troubles; and if need be, rest satisfied, will watch at your couch in the hour of affliction.

We receive you as devotees to a noble profession, and to-day consummate ties, which, we hope, will ever after be reverted to, by all, with feelings of pleasure. To those whom we meet to-day for the first time, let me again assure you of our kind intentions. We feel complimented by your choice, and hope that our intercourse for the ensuing four months, will tend to increase, rather than lessen, that confidence which has honored us with your preference.

To those of you, whose faces are familiar, and with whom we had the pleasure of laboring the past session, it is unnecessary for me to say welcome; you know our hearts, and are well apprised that our every energy has, and will continue to be, exerted for your advancement. By your return to our Institution we feel highly complimented. It is certainly a token of your regard, and an evidence, too, that your confidence has not been lessened by that intimate relation which we sustained during the past summer.

The labors which commence with this hour, and which are to continue four months, are both to student and teacher, arduous and responsible. That we may have some idea of the responsibilities which surround us, let us, for a moment, glance at the position of both—the responsibilities which the teacher assumes, and those which must necessarily surround you as physicians.

The position of a public teacher of medicine, is one of more responsibility than you would at first imagine. Did a want of success in imparting information, alone affect the teacher, the responsibility would by no means be so great; but a moment's reflection will convince all, that it is the student, and not so much the teacher, that is injured. It is here, gentlemen, that you have come to receive instruction in the various branches of the medical science; your presence here is an evidence of your confidence in the present faculty: should we betray that confidence, teaching you false and dangerous doctrines, or leave untaught important truths, we would not only be doing you a very serious wrong by affecting your prospects as physicians, but, perhaps, thousands committed to your hands would be made to suffer by the error. The position, then, is one of great responsibility—one from which there may result very great good, or a corresponding evil—the good or evil, as before suggested, not being alone confined to you as physicians, but extending itself to those who may honor you with their confidence. With such feelings in regard to our position, let me assure you, that every member of this faculty will do all in his power to impress you with those great principles in medicine, which are to direct you in the hour of trial.

But, gentlemen, you too have your responsibilities—your part to perform, and without its faithful performance, our labors, however assiduous, must fall far short of the great object to be attained. No one of you, I hope, has adopted our noble profession without a comprehensive view of the great responsibility which necessarily attends it. With a proper appreciation of the position that you must necessarily occupy as physicians, there is not one of you but must be spurred up to duty by the thought of that remorse of conscience which must result from a feeling of incompetency at the bedside of a dying patient.

In a few months, the lives of many of your fellow beings will be placed in your hands. The father, mother, child, and in fact, the whole community in which you reside, will turn to you for assistance and advice in the hour of affliction. Imagine the mother weeping over her tender and innocent babe—the wife over the prostrate husband and father, the only hope and support of a doting family; or a husband kneeling by the side of a loving wife and mother; upon your skill depends the happiness or misery of that family. Every eye is turned to you as their physician and deliverer. Your every gesture and change of feature or expression, is watched with intensity, to see, if possible, some change upon which to build a hope. You will be called upon, too, in such tones of agony and supplication, as will almost unnerve you for the emergency. This, gentlemen, is no imaginative sketch, but stern reality—scenes through which you must all pass. Say, then, that in such an hour you should feel your incompetency, and feel, too, that it was alone the result of a want of application upon your part, and the picture is complete. Your feelings under such circumstances I shall not attempt to depict—may it never be your fortune to realize them. Let me beseech you, then, to leave nothing undone that will tend to prepare you for such trying scenes. You may not notice or care for misspent time now, but the day will surely come when you will look back with regret upon every hour that has been squandered during your professional studies.

Again, let me assure you, that we shall leave nothing undone that we think will tend to advance your interests, and if we see upon your part the same manifestation, which I feel confident we will, our labors, however arduous, will be made pleasant.

As your presence here to-day is an evidence of your intention to unite your destinies with ours, it is certainly right and proper that you should, at this hour, hear something of the policy of the Institution with which you propose to connect yourselves. And this has been made doubly necessary by the fact, that many of you have heard conflicting statements in regard to the principles which direct us—statements which were known to be erroneous, but concocted and set afloat with the view of blasting our prospects.

Since the organization of the Atlanta Medical College, the greatest ambition of the Faculty has been to furnish advantages to medical students equal to those found in any other

Institution in the land; and not only this, but to establish the highest standard of requisitions for graduation which has, or shall be adopted and conformed to, by the medical colleges of the United States. But, notwithstanding this, and our efforts in many other particulars to forward the interests of the profesison, there are some, who by vain boasting, supercilious sneers, and in many other ways no less discreditable, have attempted to bring us into disrepute.

Yes, there are some who, from our infancy, have attempted our overthrow, and still continue their blows, but thanks to truth, justice, and a discriminating public, every blow that has been hurled at us, has recoiled upon our adversaries, and if facts and numbers are to be relied upon, they, and not us, have been the sufferers.

It is true, that the number of our adversaries is small, and daily, growing less; but, bitter, unscrupulous and uncompromising; and, therefore, we think it not amiss to investigate the pretended cause of this opposition, and to lay our position fully before you.

It would appear that our only sin is the *season of the year* that we have selected to hold our sessions—that in every other particular, we are all that they could desire.

Before attempting to discuss the objections urged against us upon this score, one word in regard to the feelings which we fear have prompted this opposition.

From whence do objections come? Principally, I regret to say, from a medical school in our own sunny South—from those with whom we come in direct competition, and who never until the *success* of the Atlanta Medical College was a fixed fact, saw anything wrong in summer teaching, although quite a number of summer schools had been in successful operation for a series of years—schools, too, with two annual sessions. If it is the principle they combat, why, I ask, have they never made issue with the summer schools of the North, of the existence of which, they were certainly not ignorant. Is it less objectionable in the northern States than in Georgia?

No, gentlemen; but summer schools in the northern States do not conflict with their interests.

But to the objections that have been urged against summer teaching.

First, that we cannot teach Anatomy in summer. We deem it unnecessary to say much upon this subject, as many of you have, within the past two weeks satisfied yourselves upon this point, and as all will, during the session, have an opportunity of investigating for themselves.

It is evident that those who urge this as an objection, have never visited the Atlanta Medical College during the Session.

With a large and well ventilated dissecting room, and anatomical material carefully prepared during the winter months, and stored away in alcohol, dissections, all admit, who have tried it, are equally satisfactory, and much more agreeable than upon the green subject in a necessarily heated and confined atmosphere during the winter months; and before the close of this session, I am confident that you will all bear me out in this, that we can teach Anatomy as perfectly in the summer, as at any other point, North or South, during the winter months.

Again, it is urged by those who are ever prating about medical reform, and the elevation of the standard of medical education, and whose reforms consists alone in words, as will be seen hereafter—that we shorten the time of study by summer sessions, and in this way lower the standard of medical education. They tell us that the student loses, by this plan, six months office study, which they consider essential to his qualification, and without it, (*and it must come between the two courses of lectures,*) he can never be worthy of the degree of Doctor of medicine.

How, I would ask, is it possible for us to shorten the time of study, if the published requisitions for graduation are strictly observed? If you will look over the announcements of the various medical schools you will find the following as one of the requisitions: "The student must have been engaged in the study of medicine three years under a competent instructor, &c." Some have, of late, inserted "the usual time," instead of three years. But, it will be asked, what school observes this requisition? Not one, I will answer; not even those who profess to feel so much in regard to time, and who contend that an interval of six months between the courses should be made a requisition.

However anxious they may be to extend the time of study, between the courses of lectures, they are not willing to observe this, their own published requisition, the only means, as all will admit, of making uniform the time of study.

To show you the feelings of even those who profess to feel so much concern in regard to time, I hope I will be excused if I here repeat a proposition that I made two years ago, to the *then* Dean of a flourishing institution in an adjoining State —an institution, if we judge alone by her many words, first in all medical reforms. In a conversation upon this subject, with the Dean of that Institution, he was rather violent in regard to time—objecting to summer teaching, upon the ground, that the student might, by this means, graduate a few months earlier than by the system of exclusive winter teaching. During the conversation, I remarked, that the Atlanta Medical College was disposed to take as high ground upon this subject as any other Institution in the land; and as an evidence that we were disposed, and would do anything to extend the time of study, that the Faculty of the Institution with which I was connected, would pledge themselves that no student should graduate, or be admitted to examination, who did not give satisfactory evidence of having been engaged in the study three years—the full extent of the time usually specified in the requisitions for graduation—if the school of which he was Dean would do the same.

The gentleman was not prepared to accept such a proposition, and that was the last of it.

Here is the evidence of what I asserted a moment ago, that the great pretensions, of those who oppose us, to medical reform, and the elevation of the standard of medical education consists alone in words—that when they are asked to come up to their own published requisitions, the only means of overcoming that feature of medical education, which they appear to think the most objectionable, they are not prepared to do so.

Say not to me, then, that they have alone the elevation of the profession at heart. I fear there are other motives—motives which I will not mention, but leave you to infer.

I stated a moment ago, that an interval of six months between the courses was made a requisition by those who oppose us—that without this interval, the student was not admitted, although he might have been engaged in the study of medicine three, five, or even ten, years.

There is something wrong here. They assure us that their great object is to lengthen the time of study; and here you see they admit students to become candidates who have only been engaged in the study of medicine fourteen months, and reject the application of others who have pursued the same course for three, five, or even ten, years. I would ask is this a reform? What difference does it make, when the courses of lectures are attended, whether winter or summer, with interval, or without interval, if the student, after a thorough examination, is found *worthy* of the degree.

And what of this interval of six months that is considered of such vital importance to the student—*of more importance than an unlimited period of study at any other time.* It is, I assert, regarded by the majority of students, as a kind of vacation, and I am confident that I will, by preceptors, be borne out in the assertion, that quite the majority of medical students, are less familiar with what is usually taught in medical institutions, when they return to college after the six months interval, than when they left it.

It has been asked, time and again, why this Institution adopted the summer instead of the winter months, to hold her sessions. I would state, that for a time after the organization of the Institution, the Trustees and Faculty were undecided as to whether they would make it a summer or winter school; but, after passing in review the wants of the State, and of the whole South, were unanimous in their decision in favor of the summer months.

One fact which did much to influence them in the adoption of summer sessions, was, that numbers of young gentlemen from the South were annually found in the summer schools of the Northern States.

However much they might have preferred to have remained in their own native South, from the absence of any summer school in the Southern States, they were forced to leave their homes to mingle with those whose sympathies were by no means in unison with their own, and whose taunts and insults to Southern Students have so often resulted in unpleasant and humiliating consequences.

Yes, Gentlemen, the summer months were adopted, to place the South upon an equal footing with the North—to give *Southern Students* an opportunity of attending lectures in a *Southern school*, during the summer months.

The Atlanta Medical College is the only summer school South of "Mason's and Dixon's Line," and if we are not mistaken, the only summer school in the United States, with but one session annually.

A summer school, before the organization of this Institution, was certainly a *desideratum* in the South; if there was no other evidence of the fact, our unprecedented success abundantly proves it.

Our peculiar location was another inducement to the selection of Summer sessions, as the four months that we hold our sessions, are as healthy, if not the healthiest, four months in the year. Situated, as Atlanta is, upon a spur of the Blue Ridge—the elevation dividing the waters of the Gulf and those of the Atlantic—with an altitude of eleven hundred feet above the sea, without rivers or other local causes of disease, I have no hesitation in asserting that it is the most Southern healthful region to be found in the United States. Surrounded by all that is conducive to health, the student may feel as secure from disease in Atlanta, as he would upon the loftiest peak of the Look-out.

Those formidable epidemics which occasionally so rapidly depopulate our sister cities, need never be feared in Atlanta, as Cholera and Yellow Fever never originate, nor can be propagated, in this favored region; cases of both having been brought to our city, and failed to extend to a single individual. But, if this is not sufficient to convince all of the healthfulness of our city, let the bills of mortality be consulted, and it will be found that, notwithstanding the number of hands employed in the several machine shops in the city, the mortality of Atlanta does not exceed one and a-half per cent., including rail road accidents.

These are some of the reasons which induced us to adopt Summer session, and we have never, for a moment, regretted our course, as our success is the best evidence that it is endorsed by the profession, notwithstanding the great effort that has been made in certain quarters, to misrepresent our motives.

For more than twelve months the subject of medical education has elicited unusual attention; all, as it would appear, feeling that something must be done to raise the standard of medical education—that the adoption of some uniform system that will insure this end, is essential to the well-being of the profession.

The diversity of opinion that has already been expressed in the discussion of this subject, is the most conclusive evidence of the difficulty to be overcome, and the almost impossibility of the suggestion of any uniform system, that will meet with the views and interests of the various sections of this vast extent of territory.

But, if time permitted, I am not disposed to enter the field of discussion, or attempt a review of this important subject; but would be allowed to say that those who propose to insure the qualification of the student by making requisite to graduation, *the simple absurdity*, of an interval of six months between the two courses, and thus relieve the profession of all anxiety upon the subject, must have very contracted views, as has been abundantly shown, or else are actuated by other motives than the good of the profession.

But whatever suggestions may be made that will tend to insure the desired object, will be, as has been before said, with pleasure adopted; or whether there be suggestions or not, the Faculty are determined that no student shall receive the honors of this Institution who does not come up to the standard recognized by the first medical schools in the land. For, after all, an *inquisition* upon the *knowledge* of the candidate, is the *only* way to guard the profession, and is certainly *the only test of qualification.*

Whatever may have been the advantages offered, the student, without the necessary application, can never become qualified to assume the responsible duties of the physician. And if any apply to this faculty without the requisite information, let it be distinctly understood, that they will not *squeeze* in through this Institution—that if such enter the profession with all its honors, the sin shall not be at our door. And, again, if the recipients of the honors of this Institution should ever be found engaged in unprofessional practices—such as would tend to degrade our noble profession, the trustees and faculty reserve the right to revoke the degree, and thus strip the offender who would dare tamper with his sacred calling, of all the honors previously conferred.

We would then say to those who *just now* feel so much in regard to medical reform, come on—"Show your faith by your works," and not attempt by sneers and ungenerous insinuations, to bring your competitors into disrepute.

I might say much more, but hope that sufficient has been said to give you our position, and the principles which direct us; and if I have succeeded in this particular, I am content.

Think not that the Faculty of this Institution are opposed to *true reform*, or the elevation of the standard of medical education. If it is thought best to lengthen the time of study, making it uniform, the Faculty of this Institution will observe it strictly. Lengthen the course of lectures, if thought advisable, to six or even eight months, and add two or more additional Chairs, and in this or any other improvement that will promise the elevation of the profession, we are with it heart and hand. But, let it be distinctly understood, we shall *never* submit to an *attack* under the cloak of reform—which we *know* the present movement to be—let it come from what source it may.

In conclusion, gentlemen, let me again urge you to prepare yourselves for the responsibilities of your adopted profession —the duties of which some of you are soon to assume.

Be not content with the *honors* of this or any other Institution, for *a diploma alone*, can never make you distinguished, or give you fame. If you would succeed, your aspirations must be higher. Let your motto be *onward* and *upward*, and never, for a moment falter, until you have ascended the rugged steeps of fame, and are firmly planted on its summit.

With a fixedness of purpose, and untiring perseverance, you may accomplish almost anything you desire. Onward, then, and ever remember that—

> "Perseverance is a Roman virtue,
> That wins each God-like act, and plucks success
> Even from the spear-proof crest of rugged danger."

